# Increased risk of major depressive disorder in sleep apnea patients in Taiwan

**DOI:** 10.1038/s41598-020-80759-3

**Published:** 2021-01-12

**Authors:** Chia-Min Chen, Chia-Yu Kuo, Meng-Ni Wu, Jen-Yu Hung, Chung-Yao Hsu, Ming-Ju Tsai

**Affiliations:** 1 Division of Pulmonary and Critical Care Medicine, Department of Internal Medicine, Kaohsiung Medical University Hospital, Kaohsiung Medical University, No. 100, Tz-You 1st Road, 807 Kaohsiung, Taiwan; 2 Sleep Disorders Center, Kaohsiung Medical University Hospital, Kaohsiung Medical University, Kaohsiung, Taiwan; 3 Graduate Institute of Medicine, College of Medicine, Kaohsiung Medical University, Kaohsiung, Taiwan; 4 School of Medicine, College of Medicine, Kaohsiung Medical University, Kaohsiung, Taiwan; 5 Department of Neurology, Kaohsiung Medical University Hospital, Kaohsiung Medical University, Kaohsiung, Taiwan; 6 Department of Respiratory Care, College of Medicine, Kaohsiung Medical University, Kaohsiung, Taiwan; 7 Graduate Institute of Clinical Medicine, College of Medicine, Kaohsiung Medical University, Kaohsiung, Taiwan

**Keywords:** Diseases, Medical research, Risk factors

## Abstract

The association between sleep apnea (SA) and depression had been reported in a few previous studies. However, whether SA increases the risk of major depressive disorder (MDD) has not been studied comprehensively in a large-scale study. We performed this population-based cohort study to assess the association between SA and MDD. We identified adult patients having SA from the Taiwan National Health Insurance Research Database and excluded those having MDD before SA diagnosis. Thirty control subjects were randomly selected to match to each SA patient by age and sex. Totally, 10,259 SA patients were matched to 102,590 control subjects. The incidence rate and cumulative incidence of MDD were significantly higher in SA patients than in the control subjects (both *p* < 0.0001). Multivariable Cox regression analysis showed that SA remained an independent risk factor for incident MDD after adjusting for age, sex, residency, income level, and comorbidities (hazard ratio = 2.9 [95% CI 2.8–3.1], *p* < 0.0001). In summary, SA patients have an increased risk to develop MDD. Physicians caring for SA patients must pay attention to their psychosocial health status.

## Introduction

Sleep apnea (SA) is the most common sleep disorder, which presents with repetitive cessation of breathing during sleep, usually associated with intermittent hypoxia, sleep fragmentation and daytime sleepiness^1–6^. The prevalence of SA, estimated by Wisconsin Sleep Cohort study, was higher in men than in women (24% and 9%, respectively)^7^. The diagnosis of SA is usually confirmed by nocturnal polysomnography (PSG), and the severity is usually classified by apnea–hypopnea index (AHI)^8^. Obstructive SA is the major form of SA, accounting for more than 90% of cases. The risk factors for SA include obesity, nasal congestion, alcohol, smoking, estrogen depletion in menopause, and so on^9^. SA has been associated with cardiovascular mortality and morbidity, including myocardial infarction, arrhythmia, sudden cardiac death, and heart failure^10^.

Major depressive disorder (MDD) is one of the most common psychiatric disorders, which may lead to disability and incur significant economic burden. The prevalence of depression is 4.2% in men and 8.9% in women in a German study^11^. The lifetime prevalence of MDD was found lower (about 1.2%) in Taiwan, while this might be underestimated^12,13^. In addition to depressed mood, patients may have other symptoms such as difficulty falling asleep, daytime sleepiness, poor appetite, fatigue, and low energy sensation. These symptoms may overlap the symptoms of SA. Because functional impairment from MDD may cause family and social problems, early detection and intervention are needed.

The association between SA and MDD has been reported in some previous studies. Besides the shared symptoms, both SA and depression are associated with systemic diseases such as cardiovascular diseases and diabetes mellitus^14–17^. Douglas et al. have reported a high prevalence of depressive symptoms (32–53%) in patients with snoring or SA^18^. Although previous studies had also demonstrated the association between depression and SA^19,20^, whether SA increases the risk of MDD has not been well studied in a large-scale study comprehensively. Since SA has been associated with various comorbidities, which might contribute to the development of MDD, we performed a population-based cohort study using Taiwan National Health Insurance (NHI) Research Database (NHIRD) to assess the association between SA and MDD.

## Methods

### Data sources

Since March, 1995, the Taiwan NHI has covered ambulatory care, inpatient care, dental services, and prescription drugs with a coverage rate of more than 96% of whole population of about 23 million people^1,2^. The NHIRD, containing information about reimbursement claims in the NHI system, was managed by the Taiwan National Health Research Institutes and was released with encrypted identification of patients and healthcare providers for medical research. In this study, we used Longitudinal Health Insurance Database 2010 (LHID2010), which included one million randomly sampled subjects in the NHI system in 2013 with their associated information until the end of 2010. This study has been approved by the Institutional Review Board in Kaohsiung Medical University Hospital (KMUH-IRB-EXEMPT-20130034 and KMUHIRB-EXEMPT(II)-20150068) and was exempted from obligation to gain inform consent of the study population. All the methods were carried out in accordance with relevant guidelines and regulations.

### Study population

**Figure 1:**
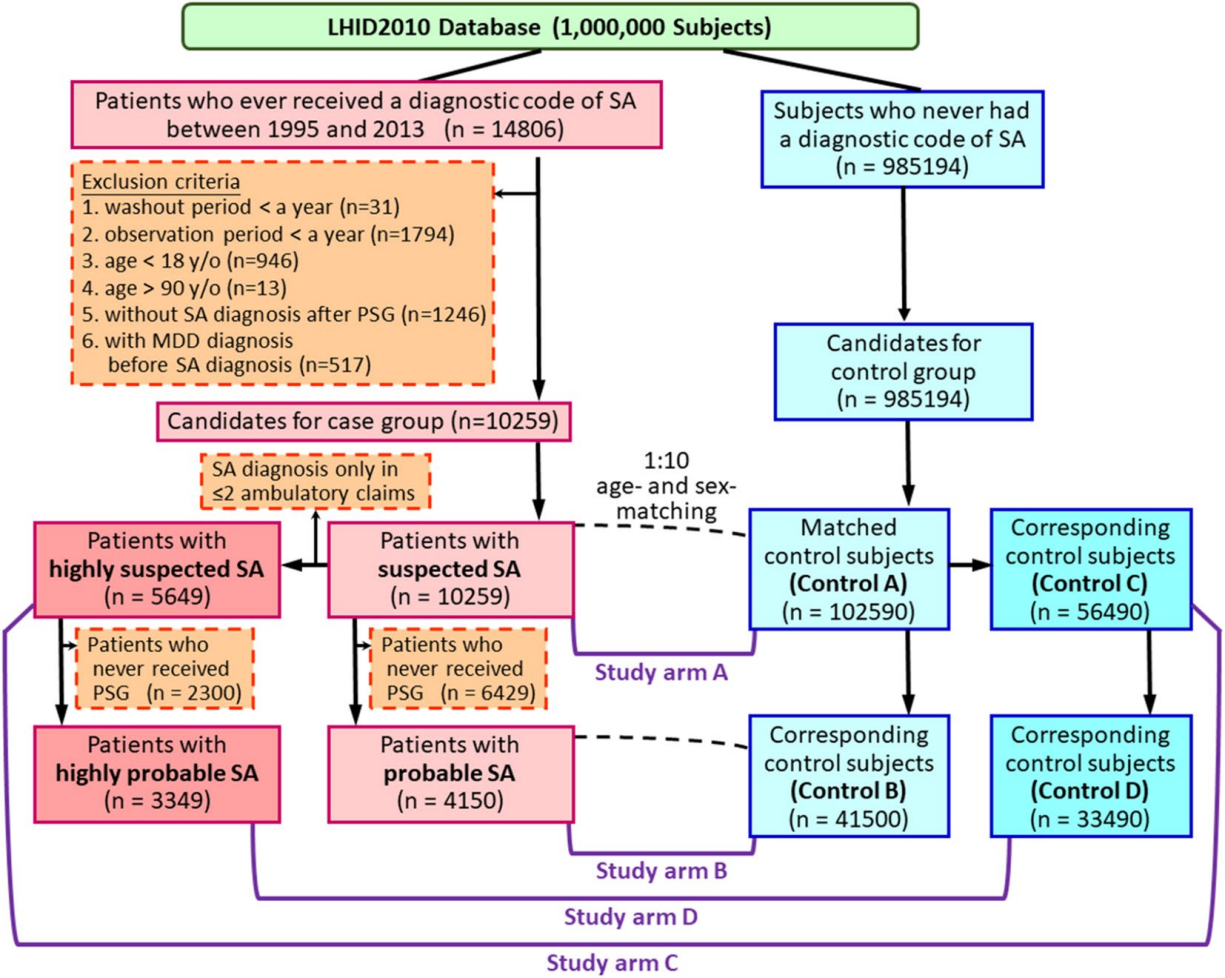
Algorithm for identifying the study population. *SA* sleep apnea, *MDD* major depressive disorder, *PSG* polysomnography.

Patients having SA diagnosis between March 1995 and December 2013 were identified initially (Fig. 1). The diagnosis of SA was identified using the International Classification of Diseases, 9th Revision, Clinical Modification (ICD-9-CM) codes of 780.51, 780.53, and 780.57^1–4^. This method for identifying SA patients in the NHIRD has been validated in previous studies^1,21–23^. The dates of their first SA diagnosis were defined as their index dates. We excluded patients with washout periods (from NHI enrollment to the index date) < 1 year or follow-up periods < 1 year to increase the likelihood of including newly-diagnosed SA cases and to ensure sufficient follow-up periods. Patients with MDD before SA diagnosis were also excluded. Besides, patients with age < 18 years or > 90 years were also excluded. The remaining SA patients (namely “suspected SA patients”) were included for further analyses.

Ten control subjects (namely “control A subjects”) were randomly selected to match to each SA patient by age and sex. The control subjects, who were assigned index dates as their corresponding SA patients, had no MDD diagnosis before their index dates and had sufficient washout periods and follow-up periods as the SA patients. The study arm A consisted of suspected SA patients and control A subjects (Fig. 1).

In order to confirm our findings, patients without SA diagnosis after PSG examination or patients who never received PSG examination and their corresponding control subjects were excluded. In another word, a subgroup of SA patients (namely “probable SA patients”), who remained having SA diagnosis after PSG, and their corresponding control subjects (namely “control B subjects”) were further extracted for another set of analyses (study arm B).

To further confirm our findings, we selected the suspected SA patients who had SA diagnosis in at least three ambulatory claims or one inpatient claim (namely “highly suspected SA patients”) and their corresponding control subjects (namely “control C subjects”) for another set of analyses (study arm C) (Fig. 1). Similarly, we also selected the probable SA patients who had SA diagnosis in at least three ambulatory claims or one inpatient claim (namely “highly probable SA patients”) and their corresponding control subjects (namely “control D subjects”) for another set of analyses (study arm D).

### Criteria and definitions of variables

Comorbidities were identified by the presence of any corresponding diagnostic codes in at least three ambulatory claims or one inpatient claim and the first appearance before the index date. Based on the comorbidities, the Charlson Comorbidity Index (CCI) score was calculated^24^.

### Study outcome

The endpoint of this study was incident MDD, defined by the first MDD diagnosis. The diagnosis of MDD was identified with the ICD-9-CM codes of 296.2 and 296.3^25^, while only the patients having MDD diagnosis in at least three ambulatory claims or one inpatient claim were considered having MDD to increase the reliability of the diagnosis.

All subjects were followed from their index dates to either incident MDD or end of the record due to the end of the study period, withdrawal from the NHI, or death, whichever came first.

### Statistical analysis

The demographic data and comorbidities were compared between the SA patients and the control subjects using the Student’s t test and Pearson χ^2^ test and for continuous variables and categorical variables, respectively. The MDD incidence rate was calculated as the number of incident MDD during the follow-up period divided by the total person-year. The MDD incidence rates (IR) were further compared between SA patients and the control subjects by calculating the incidence rate ratio (IRR). Under the assumption that the observed number of incident MDD followed a Poisson probability distribution, a 95% confidence interval (CI) was calculated for each IRR. Stratified analyses, by classifying the subjects with age, sex, residency, income level, or comorbidities, were also performed. Using multivariable analyses adjusting for age, sex, residency, income level, and comorbidities, the adjusted IRRs were calculated. Cumulative incidences of MDD in SA patients and control subjects were calculated and compared using Kaplan–Meier method and log-rank test. Using multivariable Cox regression analyses adjusting for sex, age, residency, income level, and comorbidities, the effect of SA on incident MDD was also assessed. Adjusted hazard ratios (HRs) are presented with 95% CI.

One might argue that the ICD-9-CM code of 296.3 (Major depressive disorder, recurrent episode) should not be used to identify incident MDD, because, by definition, a past depressive episode prior to the index episode is required to qualify for this diagnostic code. We therefore performed a set of sensitivity analyses, taking only ICD-9-CM code of 296.2 (major depressive disorder, single episode) for the outcome (incident MDD) (in another word, 296.3 was not considered as the outcome in the sensitivity analyses).

Extraction, computation, linkage, processing, and sampling, of data and statistical analyses were performed using SAS, Version 9.4 of the SAS System for Windows (SAS Institute Inc., Cary, NC, USA). A two-sided *p* value of < 0.05 was taken as the criterion for statistical significance.

## Results

Through the algorithm (Fig. 1), 10,259 “suspected SA” patients, including 4150 “probable SA” patients, were identified and matched to 102,590 “control A” subjects, including 41,500 “control B” subjects. Table 1 presented the baseline characteristics of the study cohorts. The mean (± standard deviation) ages of the study population were 47.1 (± 14.7) and 47.2 (± 13.3) years in study arms A and B, respectively. In study arm A, 64% of the study subjects were male; 79% of the study subjects were male in study arm B. Compared with the corresponding control subjects, SA patients had better economic status and more comorbidities (Table 1).

**Table 1:** Baseline characteristics of the study population.

	Study arm A			Study arm B		
	Suspected SA	Control A	*P* value	Probable SA	Control B	*P* value
N	10,259	102,590		4150	41,500	
**Sex, n (%)**						
Female	3696 (36%)	36,960 (36%)		879 (21%)	8790 (21%)	
Male	6563 (64%)	65,630 (64%)		3271 (79%)	32,710 (79%)	
Age (year), mean ± SD	47.1 ± 14.7	47.1 ± 14.7		47.2 ± 13.3	47.2 ± 13.3	
**Age (year), n (%)**						
≤ 40	3660 (36%)	36,600 (36%)		1366 (33%)	13,660 (33%)	
40 < age ≤ 50	2540 (25%)	25,400 (25%)		1126 (27%)	11,260 (27%)	
> 50	4059 (40%)	40,590 (40%)		1658 (40%)	16,580 (40%)	
**Residency, n (%)**			< 0.0001			< 0.0001
Northern Taiwan	5619 (55%)	51,909 (51%)		2423 (58%)	20,967 (51%)	
Other areas	4640 (45%)	50,681 (49%)		1727 (42%)	20,533 (49%)	
Monthly income (NT$), median (IQR)	21,900 (1249–43,900)	21,900 (1249–38,200)	< 0.0001	27,600 (1249–45,800)	21,900 (1249–42,000)	< 0.0001
**Monthly income (NT$), n (%)**			< 0.0001			< 0.0001
≤ 24,000	5791 (56%)	63,753 (62%)		2003 (48%)	24,395 (59%)	
> 24,000	4468 (44%)	38,837 (38%)		2147 (52%)	17,105 (41%)	
CCI score, mean ± SD	1.4 ± 1.8	0.9 ± 1.5	< 0.0001	1.5 ± 1.8	0.9 ± 1.5	< 0.0001
**CCI score, n (%)**			< 0.0001			< 0.0001
= 0	4166 (41%)	60,765 (59%)		1555 (37%)	24,505 (59%)	
= 1	2598 (25%)	21,171 (21%)		1080 (26%)	8802 (21%)	
≥ 2	3495 (34%)	20,654 (20%)		1515 (37%)	8193 (20%)	
**Underlying diseases, n (%)**						
Heart disease	463 (5%)	2377 (2%)	< 0.0001	209 (5%)	888 (2%)	< 0.0001
Myocardial infarction	105 (1%)	717 (1%)	0.0002	49 (1%)	312 (1%)	0.0029
Congestive heart failure	387 (4%)	1874 (2%)	< 0.0001	171 (4%)	662 (2%)	< 0.0001
Peripheral vascular disease	140 (1%)	995 (1%)	0.0001	65 (2%)	368 (1%)	< 0.0001
Major neurological disorder	1024 (10%)	5770 (6%)	< 0.0001	467 (11%)	2144 (5%)	< 0.0001
Cerebral vascular disease	974 (9%)	5433 (5%)	< 0.0001	447 (11%)	2018 (5%)	< 0.0001
Dementia	101 (1%)	624 (1%)	< 0.0001	39 (1%)	214 (1%)	0.0005
Hemiplegia	90 (1%)	663 (1%)	0.0061	43 (1%)	278 (1%)	0.0071
Chronic pulmonary disease	2765 (27%)	15,845 (15%)	< 0.0001	1187 (29%)	6128 (15%)	< 0.0001
Connective tissue disease	228 (2%)	1346 (1%)	< 0.0001	88 (2%)	455 (1%)	< 0.0001
Peptic ulcer disease	3143 (31%)	19,258 (19%)	< 0.0001	1311 (32%)	7744 (19%)	< 0.0001
Liver disease	2115 (21%)	12,376 (12%)	< 0.0001	1005 (24%)	5389 (13%)	< 0.0001
Diabetes mellitus	1282 (12%)	9452 (9%)	< 0.0001	570 (14%)	3906 (9%)	< 0.0001
Renal disease	420 (4%)	2472 (2%)	< 0.0001	169 (4%)	985 (2%)	< 0.0001
Cancer	497 (5%)	3200 (3%)	< 0.0001	202 (5%)	1205 (3%)	< 0.0001

*SA* sleep apnea, *NT$* New Taiwan Dollar, *CCI* Charlson Comorbidity Index, *SD* standard deviation, *IQR* interquartile range.

The incidence rates of MDD in suspected SA patients and probable SA patients are significantly higher than the corresponding control subjects (suspected SA patients vs. control A subjects: 4.4 vs. 1.3 per 1000 patient-year, adjusted IRR [95% CI] 2.9 [2.8–3.1], *p* < 0.0001; probable SA patients vs. control B subjects: 3.1 vs. 1.1 per 1000 patient-year, adjusted IRR [95% CI] 2.1 [2.0–2.3], *p* < 0.0001) (Table 2). In the stratified analyses of the study population stratified by age, sex, residency, income level, or the presence of any comorbidity, SA patients had a significantly higher MDD incidence rate compared with the corresponding control subjects in all strata (Table 2).

**Table 2:** Incidence rate of major depressive disorder after the index date.

	Study arm A										Study arm B									
	Suspected SA				Control A				Crude IRR [95% CI]	Adjusted IRR [95% CI]	Probable SA				Control B				Crude IRR [95% CI]	Adjusted IRR [95% CI]
	N	MDD	PY	IR	N	MDD	PY	IR			N	MDD	PY	IR	N	MDD	PY	IR		
Whole study population	10,259	271	61,599.9	4.4	102,590	804	625,463.3	1.3	3.4 [3.3–3.6]*	2.9 [2.8–3.1]*	4150	72	23,478.0	3.1	41,500	268	236,227.9	1.1	2.7 [2.5–2.9]*	2.1 [2.0–2.3]*
**Stratified analyses**																				
Sex																				
Female	3696	161	22,120.9	7.3	36,960	405	228,071.6	1.8	4.1 [3.8–4.4]*	3.5 [3.3–3.8]*	879	27	4620.3	5.8	8790	76	47,297.4	1.6	3.6 [3.1–4.2]*	2.6 [2.2–3.1]*
Male	6563	110	39,479.0	2.8	65,630	399	397,391.8	1.0	2.8 [2.6–2.9]*	2.4 [2.2–2.5]*	3271	45	18,857.6	2.4	32,710	192	188,930.5	1.0	2.3 [2.1–2.6]*	1.9 [1.7–2.1]*
Age																				
≤ 50	6200	163	38,565.5	4.2	62,000	450	391,289.1	1.2	3.7 [3.5–3.9]*	3.1 [2.9–3.3]*	2492	39	14,529.5	2.7	24,920	148	145,951.1	1.0	2.6 [2.4–2.9]*	2.1 [1.9–2.3]*
> 50	4059	108	23,034.4	4.7	40,590	354	234,174.2	1.5	3.1 [2.9–3.3]*	2.7 [2.5–2.9]*	1658	33	8948.5	3.7	16,580	120	90,276.8	1.3	2.8 [2.5–3.1]*	2.1 [1.9–2.4]*
Residents in																				
Northern Taiwan	5619	136	33,607.9	4.0	51,909	391	315,233.1	1.2	3.3 [3.1–3.5]*	2.9 [2.7–3.1]*	2423	42	14,352.1	2.9	20,967	126	118,453.9	1.1	2.8 [2.5–3.0]*	2.2 [2–2.5.0]*
Other areas	4640	135	27,992.0	4.8	50,681	413	310,230.3	1.3	3.6 [3.4–3.9]*	3.0 [2.8–3.2]*	1727	30	9125.9	3.3	20,533	142	117,774.0	1.2	2.7 [2.4–3.1]*	2.0 [1.8–2.2]*
Monthly income																				
≤ NT$24,000	5791	193	34,297.3	5.6	63,753	566	388,317.7	1.5	3.9 [3.6–4.1]*	3.2 [3.0–3.4]*	2003	42	10,840.2	3.9	24,395	172	137,753.1	1.2	3.1 [2.8–3.4]*	2.3 [2.1–2.6]*
> NT$24,000	4468	78	27,302.5	2.9	38,837	238	237,145.7	1.0	2.8 [2.6–3.1]*	2.4 [2.2–2.6]*	2147	30	12,637.7	2.4	17,105	96	98,474.9	1.0	2.4 [2.2–2.7]*	1.8 [1.6–2.0]*
Comorbidity																				
No (CCI score = 0)	4166	78	27,346.4	2.9	60,765	359	394,379.9	0.9	3.1 [2.9–3.4]*	3.2 [3.0–3.5]*	1555	14	9560.1	1.5	24,505	111	148,329.8	0.7	2.0 [1.7–2.2]*	2.0 [1.8–2.3]*
Yes (CCI score ≥ 1)	6093	193	34,253.5	5.6	41,825	445	231,083.5	1.9	2.9 [2.7–3.1]*	2.8 [2.6–2.9]*	2595	58	13,917.9	4.2	16,995	157	87,898.1	1.8	2.3 [2.1–2.6]*	2.1 [1.9–2.4]*

The adjusted IRRs were calculated by multivariable analyses adjusting for sex, age, residency, income and the presence of various comorbidities. (except for the variable used for stratification).

*p < 0.0001.

*NT$* New Taiwan Dollar, *CCI* Charlson Comorbidity Index, *N* number of patients, *MDD* major depressive disorder (number of patients), *PY* total patient-years, *IR* incident rate, as expressed as MDD incidence per 1000 patient-years, *IRR* incidence rate ratio, *CI* confidence interval.

**Figure 2:**
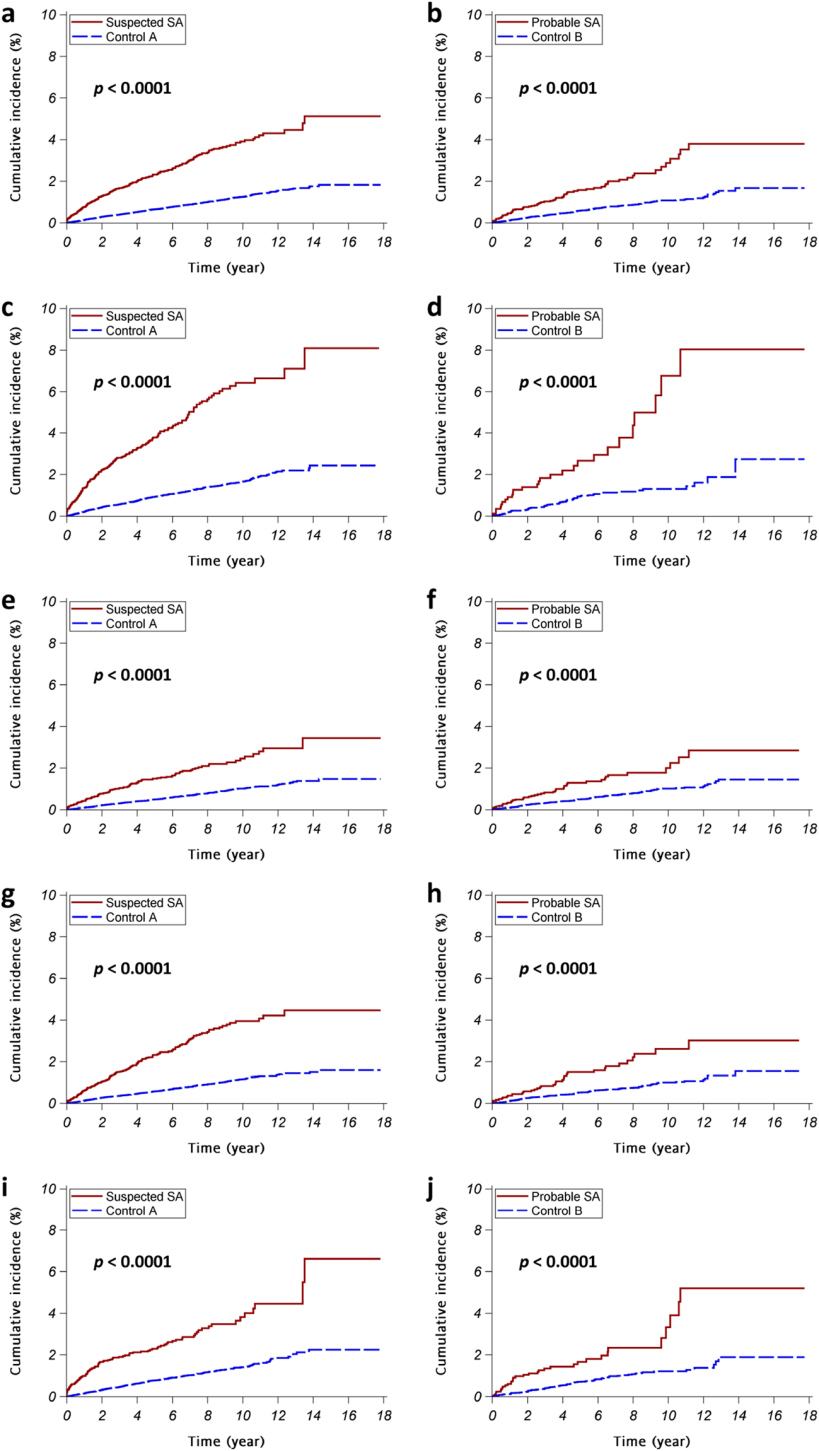
The cumulative incidences of major depressive disorder (MDD). The red continuous lines and blue dashed lines show the cumulative incidence of MDD for the sleep apnea patients and the control subjects, respectively. (**a**, **c**, **e**, **g**, **i**) suspected sleep apnea patients vs. control A subjects; (**b**, **d**, **f**, **h**, **j**) probable sleep apnea patients vs. control B subjects; (**a**, **b**) all eligible subjects; (**c**, **d**) female subjects; (**e**, **f**) male subjects; (**g**, **h**) subjects ≤ 50 years old; (**i**, **j**) subjects > 50 years old.

The cumulative MDD incidence are significantly higher in suspected SA patients and probable SA patients than in the corresponding control subjects (both *p* < 0.0001) (Fig. 2a,b). In the strata classified with sex or age, SA patients had a significantly higher cumulative MDD incidence compared with the corresponding control cohorts (all *p* < 0.0001) (Fig. 2c–j).

**Figure 3:**
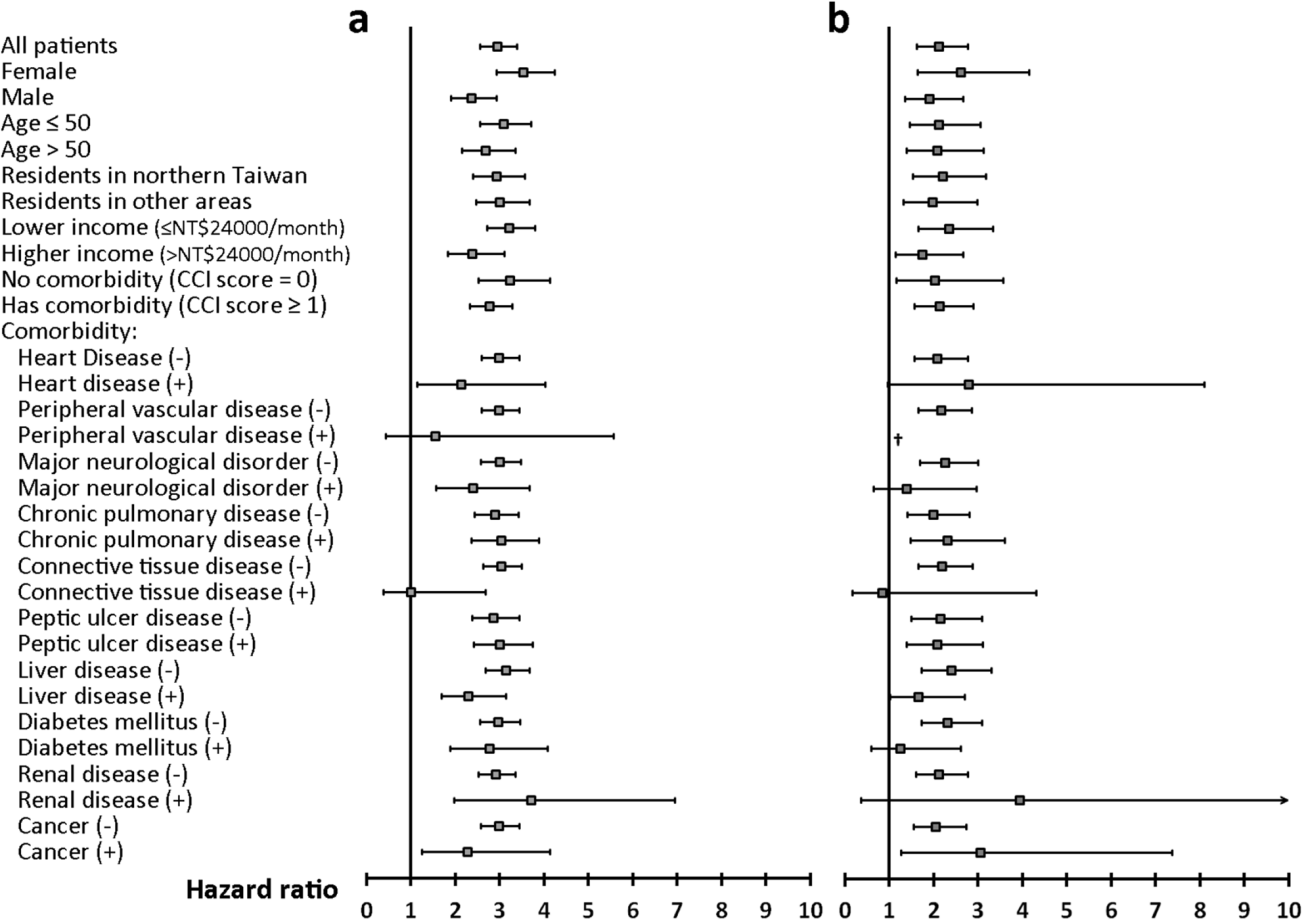
Stratified analyses of multivariable Cox regression analyses assessing the effect of sleep apnea on incident major depressive disorder. The results are presented with adjusted HRs (95% CI) of sleep apnea, which are adjusted for sex, age, residency, income level and the presence of various comorbidities (except for the variable used for stratification). (**a**) Study arm A (suspected sleep apnea patients and control A subjects); (**b**) study arm B (probable sleep apnea patients and control B subjects). *SA sleep apnea, *CCI* Charlson Comorbidity Index, HR hazard ratio, *CI* confidence interval. ^†^Due to small sample size, hazard ratio cannot be estimated.

On multivariable Cox proportional hazards regression analyses adjusted for age, sex, residency, income, and comorbidities, SA was an independent risk factor for incident MDD (study arm A: adjusted HR [95% CI] 2.9 [2.6–3.4], *p* < 0.0001; study arm B: adjusted HR [95% CI] 2.1 [1.6–2.8], *p* < 0.0001) (Fig. 3). Stratified analyses revealed that SA was associated with a higher risk for developing MDD in nearly all strata (Fig. 3).

To confirm our findings, we selected the suspected SA patients and probable SA patients who had SA diagnosis in at least three ambulatory claims or one inpatient claim (namely “highly suspected SA patients” and “highly probable SA patients”, respectively) and their corresponding control subjects (namely “control C subjects” and “control D subjects”, respectively) for another sets of analyses (study arms C and D) (Fig. 1, Supplementary Table S1 online). The study arms C and D showed consistent results as the findings in the study arms A and B (Supplementary Figs. S1, S2 and Table S2 online).

The sensitivity analyses, taking only ICD-9-CM code of 296.2 (major depressive disorder, single episode) for the outcome (incident MDD), showed similar findings as those in the previous analyses (Supplementary Figs. S3–S6 and Tables S3–S4 online).

## Discussion

In this large population-based cohort study, we found that patients with SA had a significantly higher MDD incidence than the subjects without SA. Multivariable analyses adjusting for age, sex, and comorbidities showed that SA remained an independent risk factor for developing MDD. In further investigation, the patients having SA diagnosis after PSG also had higher incidence of MDD.

A few previous studies have used NHIRD to investigate the association between SA and mood disorder. Chen et al. used data from Longitudinal Health Insurance Database 2000 (LHID2000) to compare the risk of subsequent depressive disorder during one-year follow-up in 2818 patients being diagnosed with SA after PSG and 14,090 matched control subjects^26^. They used ICD-9-CM codes 296.2, 296.3, 300.4, and 311 to identify the diagnosis of depressive disorder, whereas we used only 296.2 and 296.3 to identify pure major depressive disorder. As in our study, they showed SA as an independent risk factor for subsequent depressive disorder. Another study using LHID2000 by Lu et al. showed an increased risk of mood disorder, especially MDD and bipolar disorder, in SA patients than subjects in the comparison group^25^. They mixed MDD with bipolar disorders and unspecified episodic mood disorders, whereas we focused on MDD. Furthermore, a study using Longitudinal Health Insurance Database 2005 (LHID2005) by Pan et al. also found a bidirectional association between SA and depression^27^. As Chen et al.^26^ they used ICD-9-CM codes 296.2, 296.3, 300.4, and 311 to identify the diagnosis of depression^27^. Both Lu et al.^25^ and Pan et al.^27^ used only diagnostic codes to identify SA patients, whereas we performed comprehensive analyses using both the cohort with SA patients identified by diagnostic codes and a subtracted cohort with SA patients diagnosed after PSG. Although some differences existed in the study design, these studies consistently showed increased risk of depression in SA patients, as shown in our study.

Other studies involving different study population showed similar results as our findings. In a prospective study involving 447 people diagnosed with OSA in a German sleep center, the prevalence of depression according to ICD-10 was 21.5% in patients with AHI > 9 (n = 303)^28^. Heinzer et al. performed a general population-based study including 2121 people with polysomnography data in Switzerland. The prevalence of moderate-to-severe sleep-disordered breathing (≥ 15 events per hour) was 23.4% in women and 49.7% in man. Multivariable analysis showed the upper quartile for the apnea–hypopnea index (> 20.6 events per hour) was associated independently with depression (odds ratio = 1.92, *p* = 0.029)^29^. A population-based study in Australia showed that people with OSA and daytime sleepiness had a strong association with depression (mild–moderate apnea: adjusted odds ratio [95% CI] 3.86 [1.87–7.95]; severe apnea: adjusted odds ratio [95% CI] 4.82 [1.42–16.35])^30^.

Although some previous studies have shown the association between SA and depression, the definite mechanisms have not been fully understood^31^. Sleep fragmentation and hypoxemia might contribute to the incident MDD in SA patients. Sleep fragmentation and sleep deprivation result in excess daytime sleepiness and may worsen the cognitive function and mood^32,33^. The SA patients with excess daytime sleepiness are more likely to have depressive symptoms^34^. Elra et al. also found that daytime sleepiness, sleep medications, and initial insomnia were independently related to depression in SA patients, whereas SA severity was not^35^. Although some studies showed that intermittent hypoxia was not significantly associated with depressive symptoms^31^, others still demonstrated the possible association between hypoxia and depression. Bardwell et al. conducted a randomized controlled trial of using continuous positive airway pressure (CPAP) treatment or oxygen supplementation in SA patients with depression and found improvement in psychological symptoms in the patients receiving oxygen supplementation, but not those using CPAP treatment^36^. This finding might suggest that hypoxemia played an important role in SA-associated depression, while the actual mechanisms remained uncertain. The IR of MDD appeared lower in probable SA patients than suspected SA patients. Although information about long-term oxygen supplementation or CPAP treatment was not available in the NHIRD, we believe those having SA diagnosis after PSG might have higher chance to be adequately treated, which ameliorated the effect of SA in increasing the risk of MDD.

Neurotransmitters have important roles in both depression and SA. The majority of evidence supports under-activation of serotonin system in depression^37^. Reduced serotonin 1A receptor binding was also associated with the pathophysiological changes of depression^38^. Alteration of serotonin system affects both respiratory and sleep-awake cycle, which may contribute to SA. Loss of serotonergic inputs to laryngeal motor neurons in nucleus ambiguus compromises upper airways patency^39^. An animal study showed that serotonin 1A receptor had an important role in neural control of upper airway patency^40^.

On the other hand, inflammatory cytokines are also important mediators between SA and depression. In one meta-analysis, the levels of inflammatory markers, such as interleukin 6, interleukin 8, and tumor necrosis factor alpha were higher in SA patients^41^. Chronic exposure to these inflammatory cytokines may lead to psychiatric disorder and depression. Cytokines activate inflammatory signaling pathways in the brain and result in changes of glutamate, monoamine, and neuropeptide systems, contributing to the development of depression^42^.

In the current study, the IR of MDD were higher among female subjects than the male subjects. The HR and IRR of SA appeared higher in female subjects than in male subjects, suggesting that the effect of SA on incident MDD might be more prominent in the female. The female had greater proinflammatory responses of monocytes induced by sleep loss^43^. The enhanced inflammatory responses might contribute to the development of mood disorders in SA patients, and the female patients might have greater effects from SA.

Out study still had some limitations. Firstly, the diagnosis of SA and MDD based on diagnostic codes might include misclassified cases. Therefore, we performed further analyses of SA patients diagnosed after PSG and found consistent results. In addition, the method using diagnostic codes to identify SA and MDD in the NHI database has been validated and used in previous studies^21–23,25^. Secondly, the symptoms, severity and treatment (CPAP or oxygenation therapy) for SA were not adequately available in the NHI database. Previous studies suggested daytime sleepiness and hypoxemia might lead to the development of MDD, so treatment of SA may theoretically decrease the risk of MDD. We believed that a proportion of SA patients included in our study had received treatment, so the risk of developing MDD might be underestimated. Nevertheless, we still found significantly increased MDD incidence in SA patients. Thirdly, some risk factors for MDD, such as alcoholism, were not available in the NHIRD, and we did not control for the use of medications, such as hypnotics, in the current study. The interpretation of our findings should be careful to account for the possible impacts from these factors. Fourthly, the index dates of SA patients and the dates of first MDD diagnosis might not be the actual onset dates. As we have no chance to identify individuals’ intervals of diagnostic delay, the interpretation of our findings should therefore be careful.

In conclusion, this large nationwide population-based cohort study confirmed SA as an independent risk factor for incident MDD. Clinicians should pay attention to any signs of MDD while seeing patients with SA.

## Supplementary Information

Supplementary Information.

## Abbreviations

AHI: Apnea–hypopnea index
CI: Confidence interval
CPAP: Continuous positive airway pressure
ICD-9-CM: International Classification of Diseases, 9th Revision, Clinical Modification
ICD-10: International Classification of Diseases, 10th Revision
HR: Hazard ratio
IQR: Interquartile range
IR: Incidence rate
IRR: Incidence rate ratio
MDD: Major depressive disorder
NHI: National Health Insurance
NHIRD: National Health Insurance Research Database
PSG: Polysomnography
SA: Sleep apnea
SD: Standard deviation
