# Supplementary material for: Increased risk of major depressive disorder in sleep apnea patients in Taiwan
Source: Sci Rep. 2021 Jan 12;11:765. doi: 10.1038/s41598-020-80759-3 (PMC7803988; doi:10.1038/s41598-020-80759-3)
Supplement: Supplementary file 1 — Supplementary Information. [file 41598_2020_80759_MOESM1_ESM.pdf]

Original Article

Increased Risk of Major Depressive Disorder in Sleep Apnea Patients in Taiwan

Supplementary Information

Authors

Chia-Min Chen, M.D. [1]

Chia-Yu Kuo, M.D. [1,2,3]

Meng-Ni Wu, M.D. [2,4,5]

Jen-Yu Hung, M.D., Ph.D. [1,3,4,7]

Chung-Yao Hsu, M.D., Ph.D. [2,4,5]

Ming-Ju Tsai, M.D., Ph.D. [1,2,3,4,6,7,*]

1 Division of Pulmonary and Critical Care Medicine, Department of Internal Medicine, Kaohsiung Medical University Hospital, Kaohsiung Medical University, Kaohsiung, Taiwan

2 Sleep Disorders Center, Kaohsiung Medical University Hospital, Kaohsiung Medical University, Kaohsiung, Taiwan

3 Graduate Institute of Medicine, College of Medicine, Kaohsiung Medical University, Kaohsiung, Taiwan

4 School of Medicine, College of Medicine, Kaohsiung Medical University, Kaohsiung, Taiwan

5 Department of Neurology, Kaohsiung Medical University Hospital, Kaohsiung Medical University, Kaohsiung, Taiwan

6 Department of Respiratory Care, College of Medicine, Kaohsiung Medical University, Kaohsiung, Taiwan

7 Graduate Institute of Clinical Medicine, College of Medicine, Kaohsiung Medical University, Kaohsiung, Taiwan

Corresponding Author

Ming-Ju Tsai, M.D., Ph.D.

Division of Pulmonary and Critical Care Medicine, Department of Internal Medicine,
Kaohsiung Medical University Hospital, No.100, Tz-You 1st Road, 807 Kaohsiung, Taiwan
E-mail: SiegfriedTsai@gmail.com
Tel.: +886 7 3121101, ext., 5651 Fax: +886 7 3161210

Table S1. Baseline characteristics of the study population (study arms C and D).

	Study arm C			Study arm D		
	Highly suspected SA	Control C	*P* value	Highly probable SA	Control D	*P* value
**N**	5649	56490		3349	33490	
**Sex, n (%)**						
**Female**	1708 (30%)	17080 (30%)		654 (20%)	6540 (20%)	
**Male**	3941 (70%)	39410 (70%)		2695 (80%)	26950 (80%)	
**Age (year), mean ± SD**	47.2 ± 14.5	47.2 ± 14.5		47 ± 13.2	47 ± 13.2	
**Age (year), n (%)**						
**≤ 40**	1982 (35%)	19820 (35%)		1116 (33%)	11160 (33%)	
**40 < age ≤ 50**	1434 (25%)	14340 (25%)		921 (28%)	9210 (28%)	
**> 50**	2233 (40%)	22330 (40%)		1312 (39%)	13120 (39%)	
**Residency, n (%)**			<0.0001			<0.0001
**Northern Taiwan**	3162 (56%)	28586 (51%)		2014 (60%)	16958 (51%)	
**Other areas**	2487 (44%)	27904 (49%)		1335 (40%)	16532 (49%)	
**Monthly income (NT$), median (IQR)**	21900 (1249-43900)	21900 (1249-38200)	<0.0001	27600 (1249-45800)	21900 (1249-42000)	<0.0001
**Monthly income (NT$), n (%)**			<0.0001			<0.0001
**≤ 24000**	3102 (55%)	34625 (61%)		1594 (48%)	19543 (58%)	
**> 24000**	2547 (45%)	21865 (39%)		1755 (52%)	13947 (42%)	
**CCI score, mean ± SD**	1.5 ± 1.8	0.9 ± 1.5	<0.0001	1.5 ± 1.8	0.8 ± 1.5	<0.0001
**CCI score, n (%)**			<0.0001			<0.0001
**= 0**	2207 (39%)	33482 (59%)		1271 (38%)	19936 (60%)	
**= 1**	1408 (25%)	11712 (21%)		851 (25%)	7058 (21%)	
**≥ 2**	2034 (36%)	11296 (20%)		1227 (37%)	6496 (19%)	
**Underlying diseases, n (%)**						
**Heart disease**	292 (5%)	1299 (2%)	<0.0001	174 (5%)	694 (2%)	<0.0001
**Myocardial infarction**	64 (1%)	401 (1%)	0.0004	37 (1%)	245 (1%)	0.0181
**Congestive heart failure**	245 (4%)	1013 (2%)	<0.0001	143 (4%)	514 (2%)	<0.0001
**Peripheral vascular disease**	89 (2%)	555 (1%)	<0.0001	49 (1%)	289 (1%)	0.0005
**Major neurological disorder**	611 (11%)	3175 (6%)	<0.0001	366 (11%)	1700 (5%)	<0.0001
**Cerebral Vascular disease**	585 (10%)	2994 (5%)	<0.0001	350 (10%)	1602 (5%)	<0.0001
**Dementia**	57 (1%)	335 (1%)	0.0002	31 (1%)	157 (0%)	0.0004
**Hemiplegia**	61 (1%)	372 (1%)	0.0003	37 (1%)	224 (1%)	0.0041
**Chronic pulmonary disease**	1582 (28%)	8611 (15%)	<0.0001	961 (29%)	4888 (15%)	<0.0001
**Connective tissue disease**	119 (2%)	694 (1%)	<0.0001	64 (2%)	368 (1%)	<0.0001
**Peptic ulcer disease**	1769 (31%)	10535 (19%)	<0.0001	1046 (31%)	6163 (18%)	<0.0001
**Liver disease**	1196 (21%)	6914 (12%)	<0.0001	802 (24%)	4284 (13%)	<0.0001
**Diabetes mellitus**	741 (13%)	5251 (9%)	<0.0001	447 (13%)	3110 (9%)	<0.0001
**Renal disease**	248 (4%)	1382 (2%)	<0.0001	143 (4%)	791 (2%)	<0.0001
**Cancer**	308 (5%)	1694 (3%)	<0.0001	165 (5%)	938 (3%)	<0.0001

Abbreviation: SA = sleep apnea; NT$ = New Taiwan Dollar; CCI = Charlson Comorbidity Index
SD = standard deviation; IQR = interquartile range.

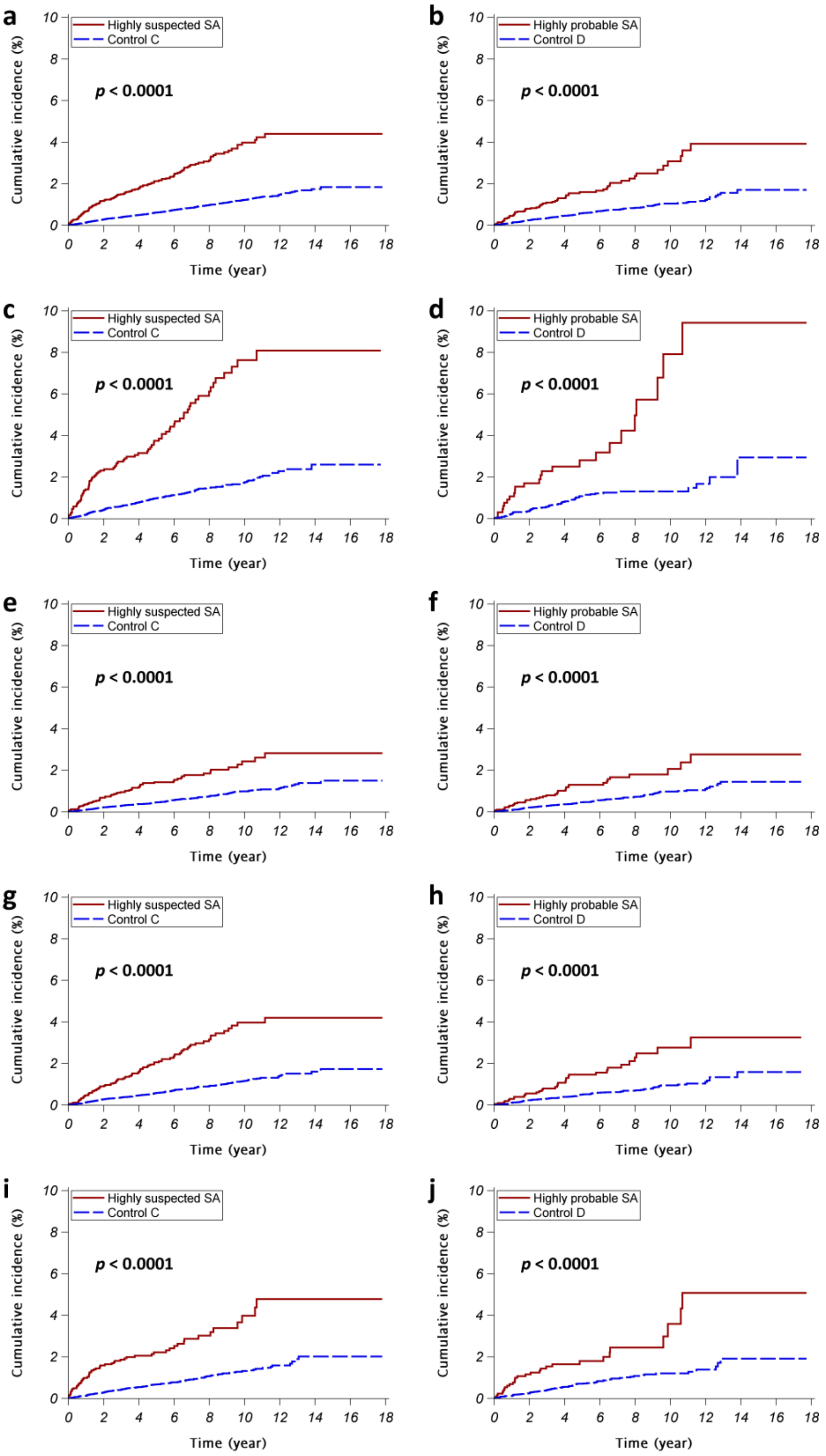

Figure S1. The cumulative incidences of major depressive disorder (MDD) (study arms C and D).

The red continuous lines and blue dashed lines show the cumulative incidence of MDD for the sleep apnea patients and the control subjects, respectively.
(a,c,e,g,i) highly suspected sleep apnea patients vs. control C subjects;
(b,d,f,h,j) highly probable sleep apnea patients vs. control D subjects;
(a,b) all eligible subjects; (c,d) female subjects; (e,f) male subjects;
(g,h) subjects ≤ 50 years old; (i,j) subjects >50 years old.

Table S2. Incidence rate of major depressive disorder after the index date (study arms C and D).

	Study arm C										Study arm D									
	Highly suspected SA				Control C				Crude IRR [95% CI]	Adjusted IRR [95% CI]	Highly probable SA				Control D				Crude IRR [95% CI]	Adjusted IRR [95% CI]
	N	MDD	PY	IR	N	MDD	PY	IR			N	MDD	PY	IR	N	MDD	PY	IR		
**Whole study population**	5649	142	34452.3	4.1	56490	433	349098.7	1.2	3.3 [3.1-3.5]*	2.8 [2.7-3.0]*	3349	61	19382.6	3.1	33490	216	195261	1.1	2.8 [2.6-3.1]*	2.3 [2.1-2.5]*
**Stratified analyses**																				
**Sex**																				
**Female**	1708	80	10105.9	7.9	17080	197	104385.3	1.9	4.2 [3.8-4.6]*	3.6 [3.3-4.0]*	654	24	3537.5	6.8	6540	65	36356.6	1.8	3.8 [3.2-4.5]*	2.8 [2.3-3.3]*
**Male**	3941	62	24346.4	2.5	39410	236	244713.4	1.0	2.6 [2.4-2.9]*	2.2 [2.1-2.4]*	2695	37	15845.1	2.3	26950	151	158904.4	1.0	2.5 [2.2-2.7]*	2.0 [1.8-2.2]*
**Age**																				
**≤ 50**	3416	84	21620.6	3.9	34160	256	218658.5	1.2	3.3 [3.1-3.6]*	2.7 [2.5-2.9]*	2037	33	12115.9	2.7	20370	118	121803.3	1.0	2.8 [2.5-3.1]*	2.2 [2.0-2.5]*
**> 50**	2233	58	12831.8	4.5	22330	177	130440.2	1.4	3.3 [3.0-3.7]*	2.8 [2.6-3.1]*	1312	28	7266.7	3.9	13120	98	73457.7	1.3	2.9 [2.5-3.3]*	2.2 [1.9-2.5]*
**Residents in**																				
**Northern Taiwan**	3162	72	19298.3	3.7	28586	213	175929.3	1.2	3.1 [2.8-3.4]*	2.8 [2.6-3.1]*	2014	35	12099.0	2.9	16958	106	98298.4	1.1	2.7 [2.4-3.0]*	2.2 [2.0-2.5]*
**Other areas**	2487	70	15154.0	4.6	27904	220	173169.4	1.3	3.6 [3.3-4.0]*	2.9 [2.7-3.2]*	1335	26	7283.5	3.6	16532	110	96962.6	1.1	3.1 [2.8-3.6]*	2.3 [2.1-2.7]*
**Monthly income**																				
**≤ NT$24000**	3102	97	18643.0	5.2	34625	289	213337.5	1.4	3.8 [3.6-4.2]*	3.1 [2.9-3.4]*	1594	35	8872.5	3.9	19543	133	113024.4	1.2	3.4 [3.0-3.8]*	2.6 [2.3-2.9]*
**> NT$24000**	2547	45	15809.3	2.8	21865	144	135761.2	1.1	2.7 [2.4-3.0]*	2.3 [2.1-2.5]*	1755	26	10510.1	2.5	13947	83	82236.7	1.0	2.5 [2.2-2.8]*	1.8 [1.6-2.1]*
**Comorbidity**																				
**No (CCI score = 0)**	2207	41	14992.1	2.7	33482	205	221343.1	0.9	3.0 [2.7-3.2]*	3.0 [2.7-3.3]*	1271	12	8020.1	1.5	19936	92	123553.8	0.7	2.0 [1.7-2.3]*	2.1 [1.8-2.4]*
**Yes (CCI score ≥ 1)**	3442	101	19460.2	5.2	23008	228	127755.6	1.8	2.9 [2.7-3.2]*	2.7 [2.5-2.9]*	2078	49	11362.5	4.3	13554	124	71707.3	1.7	2.5 [2.2-2.8]*	2.3 [2.1-2.6]*

The adjusted IRRs were calculated by multivariable analyses adjusting for sex, age, residency, income and the presence of various comorbidities (except for the variable used for stratification).

*$p<0.0001$

Abbreviation: NT$ = New Taiwan Dollar; CCI = Charlson Comorbidity Index;
N = number of patients; MDD = major depressive disorder (number of patients); PY = total patient-years;
IR = incident rate, as expressed as MDD incidence per 1000 patient-years;
IRR = incidence rate ratio; CI = confidence interval.

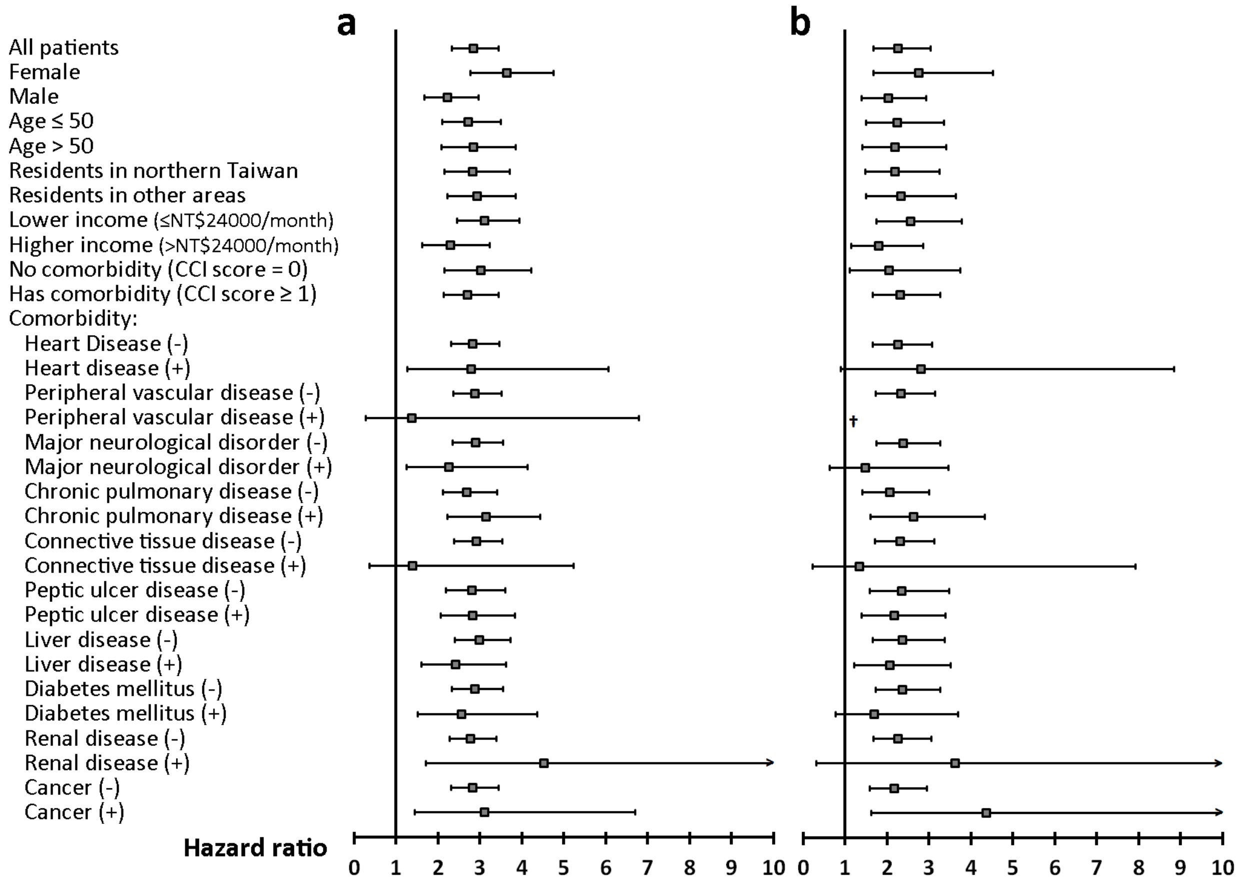

Figure S2. Stratified analyses of multivariable Cox regression analyses assessing the effect of sleep apnea on incident major depressive disorder (study arms C and D).

The results are presented with adjusted HRs (95% CI) of sleep apnea, which are adjusted for sex, age, residency, income level and the presence of various comorbidities (except for the variable used for stratification).

(a) study arm C (highly suspected sleep apnea patients and control C subjects);

(b) study arm D (highly probable sleep apnea patients and control D subjects).

*Abbreviations: SA = sleep apnea; CCI = Charlson Comorbidity Index; HR = hazard ratio; CI = confidence interval.

†: Due to small sample size, hazard ratio cannot be estimated.

Sensitivity Analyses

The following supplementary figures (Figures S3, S4, S5, S6) and tables (Tables S3, S4) are the results of analyses taking only ICD-9-CM code of **296.2 (major depressive disorder, single episode)** for the outcome (incident major depressive disorder [MDD]) (in another word, 296.3 was not considered as the outcome in the following analyses).

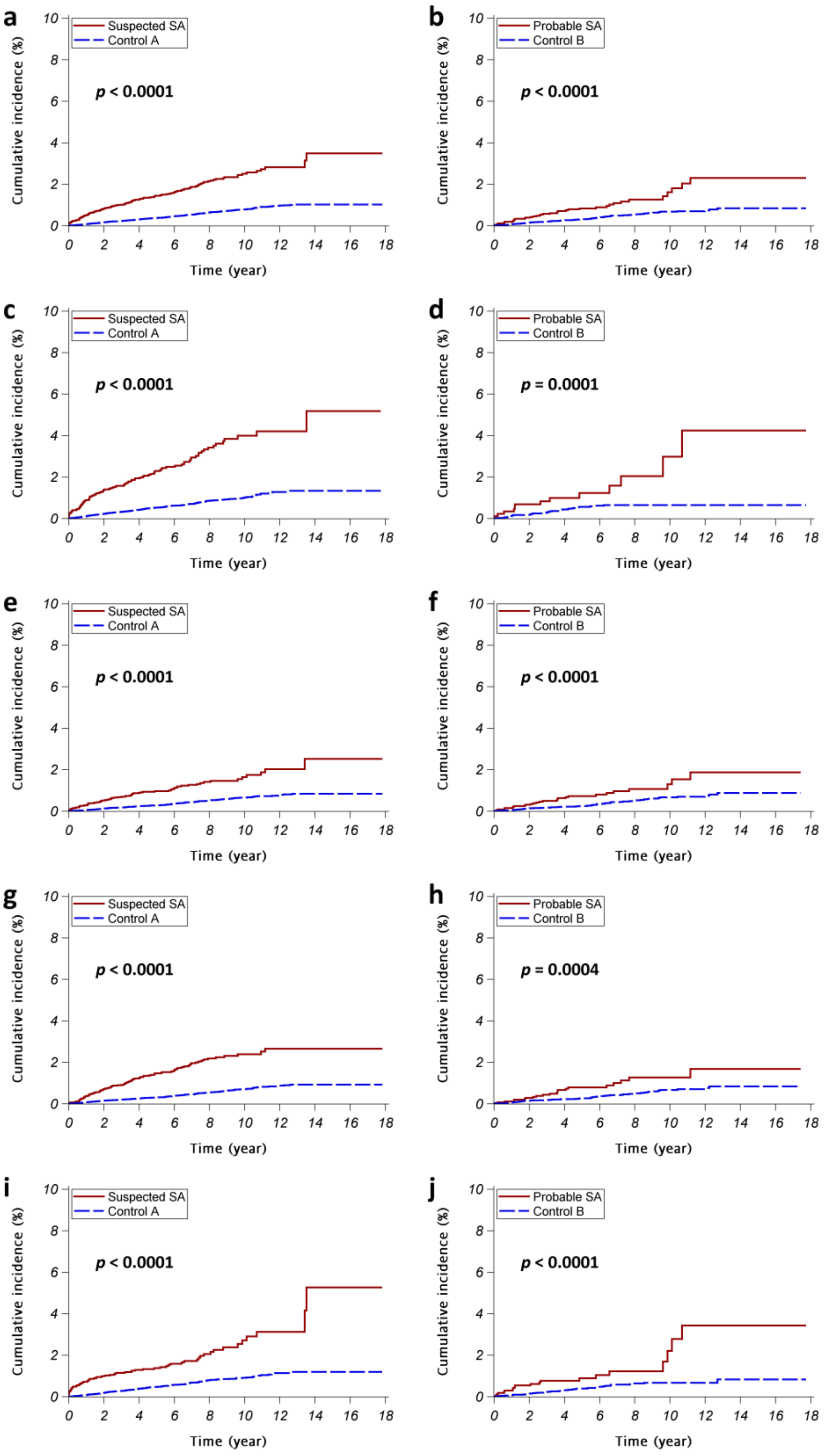

Figure S3. The cumulative incidences of major depressive disorder (MDD) (sensitivity analyses, study arms A and B).

The red continuous lines and blue dashed lines show the cumulative incidence of MDD for the sleep apnea patients and the control subjects, respectively.
(a,c,e,g,i) suspected sleep apnea patients vs. control A subjects;
(b,d,f,h,j) probable sleep apnea patients vs. control B subjects;
(a,b) all eligible subjects; (c,d) female subjects; (e,f) male subjects;
(g,h) subjects ≤ 50 years old; (i,j) subjects >50 years old.

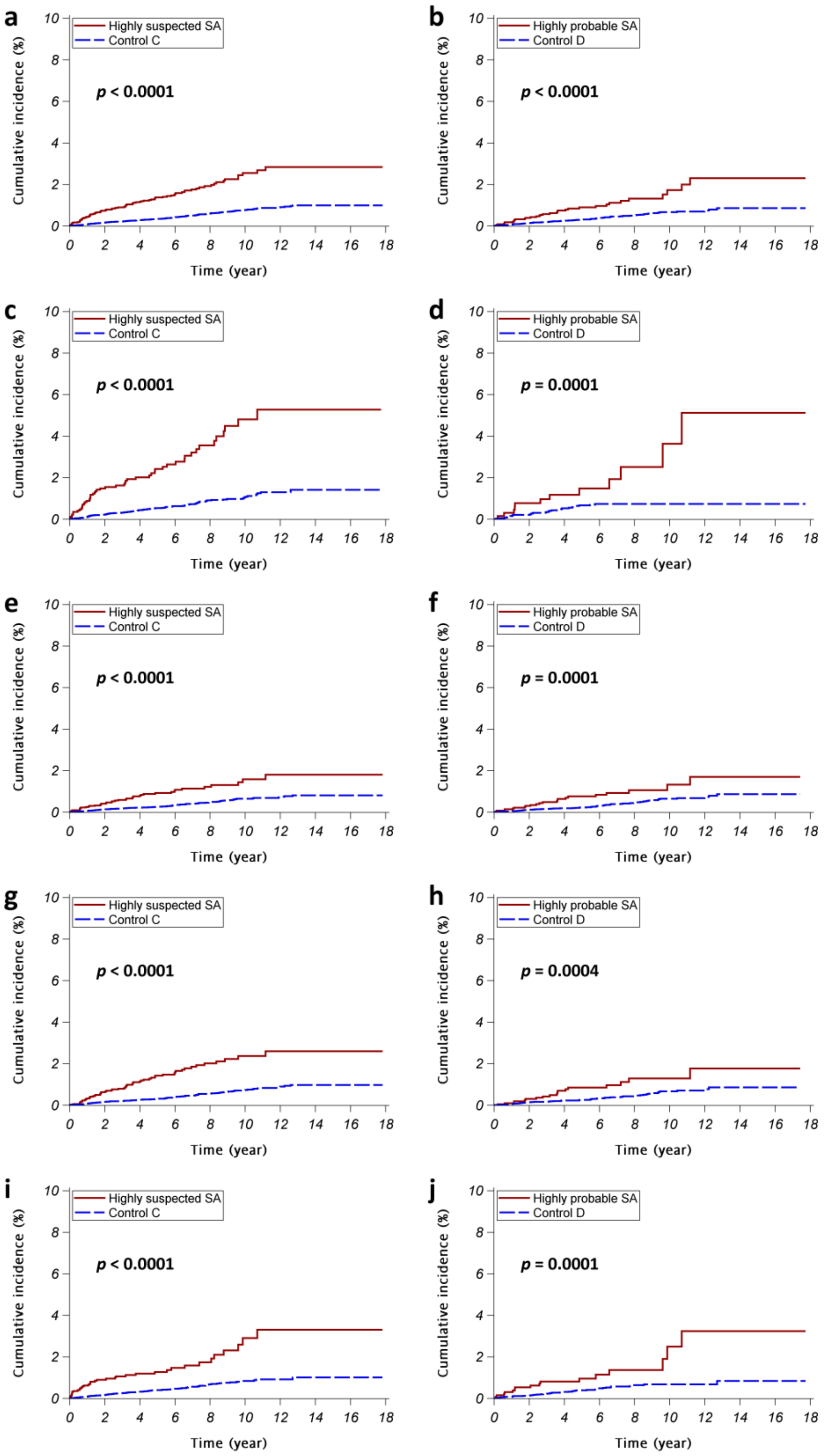

Figure S4. The cumulative incidences of major depressive disorder (MDD) (sensitivity analyses, study arms C and D).

The red continuous lines and blue dashed lines show the cumulative incidence of MDD for the sleep apnea patients and the control subjects, respectively.
(a,c,e,g,i) highly suspected sleep apnea patients vs. control C subjects;
(b,d,f,h,j) highly probable sleep apnea patients vs. control D subjects;
(a,b) all eligible subjects; (c,d) female subjects; (e,f) male subjects;
(g,h) subjects ≤ 50 years old; (i,j) subjects >50 years old.

Table S3. Incidence rate of major depressive disorder after the index date (sensitivity analyses, study arms A and B).

	Study arm A										Study arm B									
	Suspected SA				Control A				Crude IRR [95% CI]	Adjusted IRR [95% CI]	Probable SA				Control B				Crude IRR [95% CI]	Adjusted IRR [95% CI]
	N	MDD	PY	IR	N	MDD	PY	IR			N	MDD	PY	IR	N	MDD	PY	IR		
**Whole study population**	10259	172	62097.3	2.8	102590	490	626922.7	0.8	3.5 [3.4-3.7]*	3.1 [3.0-3.3]*	4150	40	23636.8	1.7	41500	157	236686.0	0.7	2.6 [2.4-2.8]*	2.1 [1.9-2.3]*
**Stratified analyses**																				
**Sex**																				
**Female**	3696	98	22446.0	4.4	36960	240	228863.9	1.0	4.2 [3.9-4.5]*	3.7 [3.4-4.0]*	879	13	4698.7	2.8	8790	41	47423.9	0.9	3.2 [2.7-3.8]*	2.4 [2.0-2.8]*
**Male**	6563	74	39651.4	1.9	65630	250	398058.8	0.6	3.0 [2.8-3.2]*	2.6 [2.5-2.8]*	3271	27	18938.1	1.4	32710	116	189262.1	0.6	2.3 [2.1-2.6]*	2.0 [1.8-2.2]*
**Age**																				
**≤ 50**	6200	102	38863.5	2.6	62000	269	392149.8	0.7	3.8 [3.6-4.1]*	3.3 [3.1-3.5]*	2492	21	14610.3	1.4	24920	91	146187.1	0.6	2.3 [2.1-2.6]*	2.0 [1.8-2.2]*
**> 50**	4059	70	23233.9	3.0	40590	221	234773.0	0.9	3.2 [3.0-3.5]*	2.9 [2.7-3.1]*	1658	19	9026.5	2.1	16580	66	90499.0	0.7	2.9 [2.6-3.3]*	2.2 [2.0-2.5]*
**Residents in**																				
**Northern Taiwan**	5619	90	33829.5	2.7	51909	241	315952.0	0.8	3.5 [3.3-3.7]*	3.2 [3.0-3.4]*	2423	27	14422.8	1.9	20967	71	118684.4	0.6	3.1 [2.8-3.5]*	2.7 [2.5-3.0]*
**Other areas**	4640	82	28267.8	2.9	50681	249	310970.7	0.8	3.6 [3.4-3.9]*	3.1 [2.9-3.3]*	1727	13	9213.9	1.4	20533	86	118001.7	0.7	1.9 [1.7-2.2]*	1.4 [1.3-1.7]*
**Monthly income**																				
**≤ NT$24000**	5791	121	34673.2	3.5	63753	354	389340.6	0.9	3.8 [3.6-4.1]*	3.2 [3.1-3.4]*	2003	24	10933.5	2.2	24395	109	138018.7	0.8	2.8 [2.5-3.1]*	2.1 [1.8-2.3]*
**> NT$24000**	4468	51	27424.1	1.9	38837	136	237582.1	0.6	3.2 [3.0-3.5]*	2.9 [2.7-3.1]*	2147	16	12703.3	1.3	17105	48	98667.3	0.5	2.6 [2.3-2.9]*	2.2 [1.9-2.4]*
**Comorbidity**																				
**No (CCI score = 0)**	4166	55	27461.9	2.0	60765	223	395058.1	0.6	3.5 [3.3-3.8]*	3.7 [3.4-3.9]*	1555	10	9580.0	1.0	24505	66	148548.5	0.4	2.3 [2.1-2.7]*	2.5 [2.2-2.8]*
**Yes (CCI score ≥ 1)**	6093	117	34635.5	3.4	41825	267	231864.6	1.2	2.9 [2.7-3.1]*	2.8 [2.6-3.0]*	2595	30	14056.8	2.1	16995	91	88137.5	1.0	2.1 [1.8-2.3]*	2.0 [1.8-2.2]*

The adjusted IRRs were calculated by multivariable analyses adjusting for sex, age, residency, income and the presence of various comorbidities (except for the variable used for stratification).

*p<0.0001

Abbreviation: NT$ = New Taiwan Dollar; CCI = Charlson Comorbidity Index;
N = number of patients; MDD = major depressive disorder (number of patients); PY = total patient-years;
IR = incident rate, as expressed as MDD incidence per 1000 patient-years;
IRR = incidence rate ratio; CI = confidence interval.

Table S4. Incidence rate of major depressive disorder after the index date (sensitivity analyses, study arms C and D).

	Study arm C										Study arm D									
	Highly suspected SA				Control C				Crude IRR [95% CI]	Adjusted IRR [95% CI]	Highly probable SA				Control D				Crude IRR [95% CI]	Adjusted IRR [95% CI]
	N	MDD	PY	IR	N	MDD	PY	IR			N	MDD	PY	IR	N	MDD	PY	IR		
**Whole study population**	5649	90	34689.0	2.6	56490	260	349852.2	0.7	3.5 [3.3-3.7]*	3.0 [2.9-3.2]*	3349	34	19520.6	1.7	33490	127	195617.2	0.6	2.7 [2.5-2.9]*	2.3 [2.1-2.5]*
**Stratified analyses**																				
**Sex**																				
**Female**	1708	50	10239.7	4.9	17080	113	104768.7	1.1	4.5 [4.1-5.0]*	4.0 [3.6-4.5]*	654	12	3609.1	3.3	6540	36	36458.4	1.0	3.4 [2.8-4.1]*	2.5 [2.1-3.0]*
**Male**	3941	40	24449.3	1.6	39410	147	245083.5	0.6	2.7 [2.5-3.0]*	2.4 [2.2-2.6]*	2695	22	15911.6	1.4	26950	91	159158.8	0.6	2.4 [2.2-2.7]*	2.2 [2.0-2.4]*
**Age**																				
**≤ 50**	3416	54	21749.6	2.5	34160	154	219108.1	0.7	3.5 [3.3-3.8]*	3.0 [2.8-3.2]*	2037	18	12184.4	1.5	20370	73	121976.1	0.6	2.5 [2.2-2.8]*	2.2 [2.0-2.5]*
**> 50**	2233	36	12939.5	2.8	22330	106	130744.1	0.8	3.4 [3.1-3.8]*	3.0 [2.7-3.3]*	1312	16	7336.2	2.2	13120	54	73641.1	0.7	3.0 [2.6-3.4]*	2.3 [2.0-2.7]*
**Residents in**																				
**Northern Taiwan**	3162	48	19389.2	2.5	28586	130	176295.1	0.7	3.4 [3.1-3.7]*	3.2 [3.0-3.5]*	2014	23	12155.2	1.9	16958	59	98494.9	0.6	3.2 [2.8-3.5]*	2.9 [2.6-3.3]*
**Other areas**	2487	42	15299.8	2.7	27904	130	173557.1	0.7	3.7 [3.3-4.0]*	2.9 [2.7-3.2]*	1335	11	7365.5	1.5	16532	68	97122.3	0.7	2.1 [1.8-2.5]*	1.6 [1.4-1.9]*
**Monthly income**																				
**≤ NT$24000**	3102	60	18809.8	3.2	34625	184	213802.2	0.9	3.7 [3.4-4.0]*	3.0 [2.8-3.2]*	1594	20	8950.6	2.2	19543	85	113212.3	0.8	3.0 [2.6-3.4]*	2.2 [2.0-2.5]*
**> NT$24000**	2547	30	15879.2	1.9	21865	76	136050.0	0.6	3.4 [3.1-3.7]*	3.1 [2.8-3.5]*	1755	14	10570.1	1.3	13947	42	82404.9	0.5	2.6 [2.0.3-3]*	2.2 [2.0-2.5]*
**Comorbidity**																				
**No (CCI score = 0)**	2207	28	15042.0	1.9	33482	126	221736.5	0.6	3.3 [3.0-3.6]*	3.3 [3.0-3.7]*	1271	9	8033.5	1.1	19936	55	123733.4	0.4	2.5 [2.2-2.9]*	2.6 [2.3-3.0]*
**Yes (CCI score ≥ 1)**	3442	62	19647.1	3.2	23008	134	128115.7	1.0	3.0 [2.8-3.3]*	2.9 [2.6-3.1]*	2078	25	11487.1	2.2	13554	72	71883.9	1.0	2.2 [1.9-2.5]*	2.1 [1.9-2.4]*

The adjusted IRRs were calculated by multivariable analyses adjusting for sex, age, residency, income and the presence of various comorbidities (except for the variable used for stratification).

**p*<0.0001

Abbreviation: NT$ = New Taiwan Dollar; CCI = Charlson Comorbidity Index;
N = number of patients; MDD = major depressive disorder (number of patients); PY = total patient-years;
IR = incident rate, as expressed as MDD incidence per 1000 patient-years;
IRR = incidence rate ratio; CI = confidence interval.

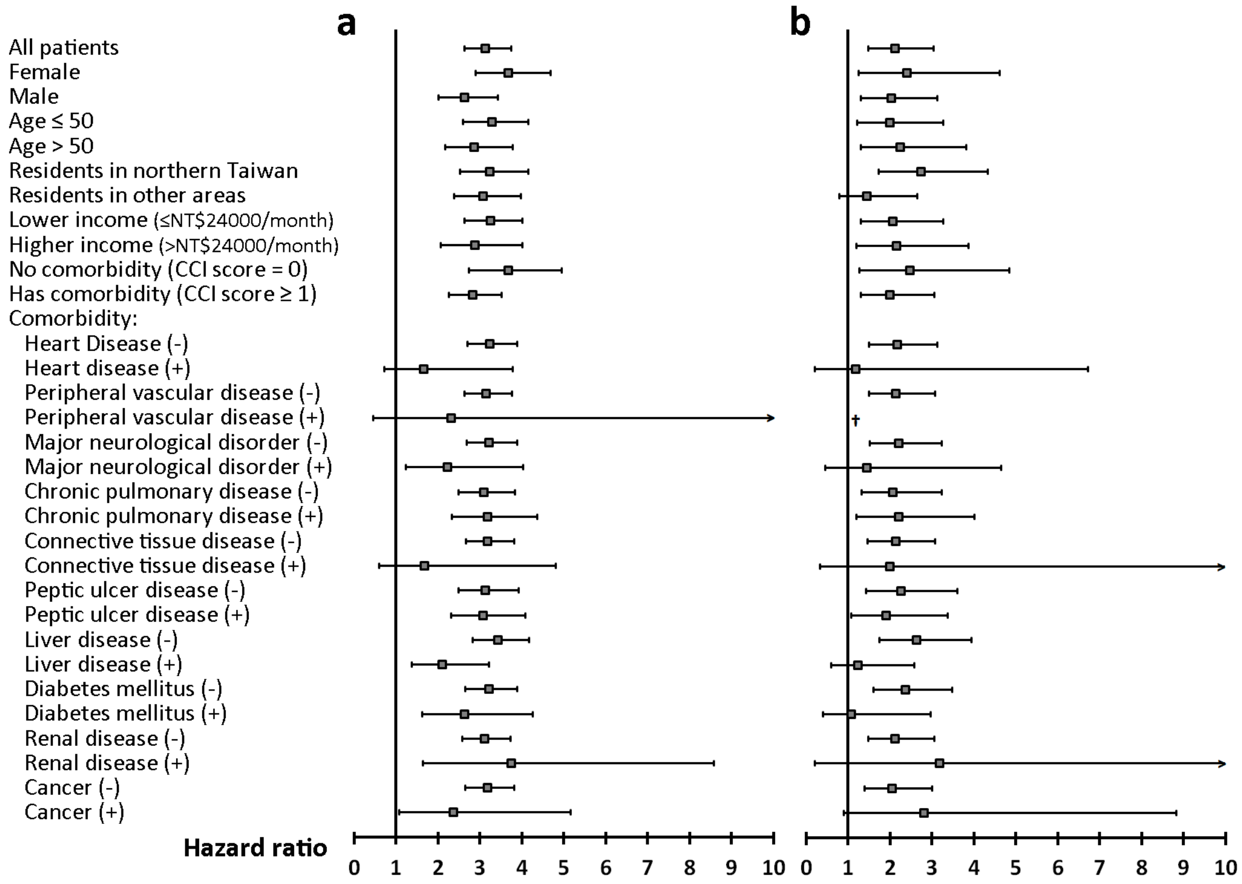

Figure S5. Stratified analyses of multivariable Cox regression analyses assessing the effect of sleep apnea on incident major depressive disorder (sensitivity analyses, study arms A and B).

The results are presented with adjusted HRs (95% CI) of sleep apnea, which are adjusted for sex, age, residency, income level and the presence of various comorbidities (except for the variable used for stratification).

(a) study arm A (suspected sleep apnea patients and control A subjects);

(b) study arm B (probable sleep apnea patients and control B subjects).

*Abbreviations: SA = sleep apnea; CCI = Charlson Comorbidity Index;
HR = hazard ratio; CI = confidence interval.

†: Due to small sample size, hazard ratio cannot be estimated.

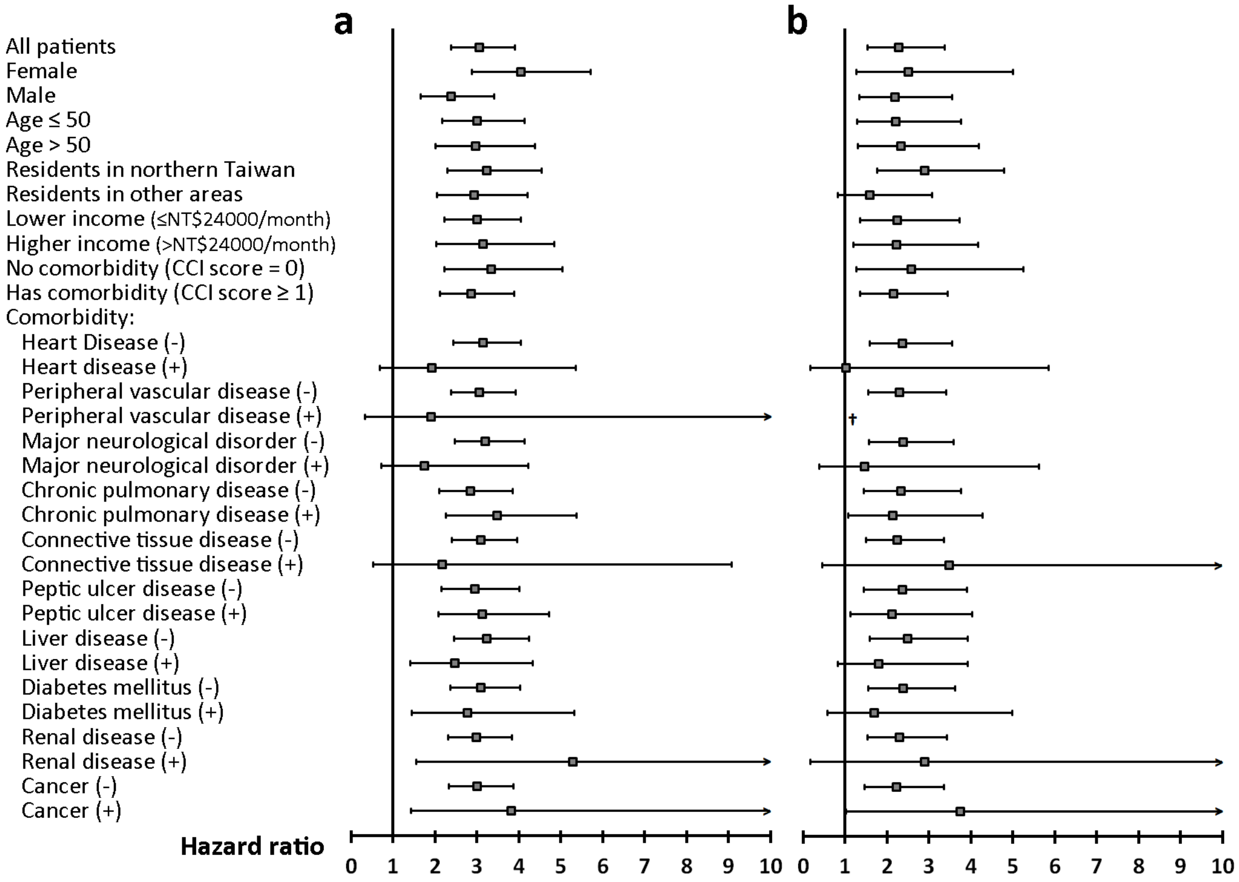

Figure S6. Stratified analyses of multivariable Cox regression analyses assessing the effect of sleep apnea on incident major depressive disorder (sensitivity analyses, study arms C and D).

The results are presented with adjusted HRs (95% CI) of sleep apnea, which are adjusted for sex, age, residency, income level and the presence of various comorbidities (except for the variable used for stratification).

(a) study arm C (highly suspected sleep apnea patients and control C subjects);
(b) study arm D (highly probable sleep apnea patients and control D subjects).

*Abbreviations: SA = sleep apnea; CCI = Charlson Comorbidity Index;
HR = hazard ratio; CI = confidence interval.

†: Due to small sample size, hazard ratio cannot be estimated.
